# Crosstalk between Gut Microbiota and Bile Acids in Cholestatic Liver Disease

**DOI:** 10.3390/nu15102411

**Published:** 2023-05-22

**Authors:** Qingmiao Shi, Xin Yuan, Yifan Zeng, Jinzhi Wang, Yaqi Zhang, Chen Xue, Lanjuan Li

**Affiliations:** State Key Laboratory for Diagnosis and Treatment of Infectious Diseases, National Clinical Research Center for Infectious Diseases, National Medical Center for Infectious Diseases, Collaborative Innovation Center for Diagnosis and Treatment of Infectious Diseases, The First Affiliated Hospital, Zhejiang University School of Medicine, Hangzhou 310003, China

**Keywords:** bile acid, gut microbiota, cholestatic liver disease, crosstalk

## Abstract

Emerging evidence suggests the complex interactions between gut microbiota and bile acids, which are crucial end products of cholesterol metabolism. Cholestatic liver disease is characterized by dysfunction of bile production, secretion, and excretion, as well as excessive accumulation of potentially toxic bile acids. Given the importance of bile acid homeostasis, the complex mechanism of the bile acid–microbial network in cholestatic liver disease requires a thorough understanding. It is urgent to summarize the recent research progress in this field. In this review, we highlight how gut microbiota regulates bile acid metabolism, how bile acid pool shapes the bacterial community, and how their interactions contribute to the pathogenesis of cholestatic liver disease. These advances might provide a novel perspective for the development of potential therapeutic strategies that target the bile acid pathway.

## 1. Introduction

In recent years, the contribution of gut microbes to health and disease has gained increasing recognition [1,2]. Diverse and complex microbial communities, including multifarious symbiotic bacteria and other microorganisms, are prevalent in the human intestine; these are collectively referred to as “gut microbiota” [3,4]. As many as 100 trillion microbes may comprise the gut microbiota, with Firmicutes and Bacteroidetes making up more than 90% of the gut microbiota [5]. The gut microbiota plays critical roles in barrier functions, metabolic functions, immune regulation, etc. [6]. For instance, the microorganisms cooperate with the host to produce numerous metabolites and signaling factors, which participate in digestion and metabolism in the host [7,8]. Some studies suggest that gut microbiota, because it can produce secondary bile acids (BAs), can be thought of as an “endocrine organ” that can influence the physiological state of the host [9].

BAs are vital metabolites of gut microbiota, and their biotransformation is closely associated with gut microbiota. The liver converts cholesterol into BAs, which are then secreted into the intestinal lumen through the biliary tract as either taurine or glycine conjugates [10,11]. In the duodenum, BAs are primarily involved in the digestion and absorption of lipids, whereas, in the ileum and colon, BAs are biotransformed under the influence of the gut microbiota [12] and are subsequently reabsorbed into the enterohepatic circulation. Dysregulation of BA homeostasis can affect the richness, composition, and metabolic activity of the intestinal microbiota, thereby influencing the development of multiple diseases such as inflammatory bowel disease (IBD), liver diseases, metabolic diseases, *Clostridium difficile* infection, and tumors [10,13,14,15,16,17,18]. Gut microbiota plays a crucial role in the metabolism of primary BAs and contributes to the diversity of the BA pool by inducing various modifications, such as deconjugation and dihydroxylation [19]. Moreover, the intestinal microbiota can affect BA synthesis and host metabolism by modulating signal transduction pathways such as farnesoid X receptor (FXR) and transmembrane G protein-coupled receptor 5 (TGR5) pathways. In turn, BAs and their receptors regulate the gut microbiota [20,21,22,23,24]. These findings suggest complex interactions between the gut microbiota and BAs.

Cholestatic liver disease (CLD) is a term including a class of hepatobiliary diseases in which the production, secretion, and excretion of bile are impaired owing to various factors, such as immunological factors, genetic factors, pharmaceutical factors, and environmental influences, preventing BAs from entering the duodenum [25]. CLD primarily includes primary biliary cholangitis (PBC), primary sclerosing cholangitis (PSC), and obstructive cholestasis [26]. The pathogenesis of PBC and PSC primarily involves immunological and genetic factors, and they are often accompanied by other autoimmune diseases such as psoriasis, rheumatoid arthritis, and IBD [27]. Ursodeoxycholic acid (UDCA) is the first-line medication for the treatment of CLD; however, ongoing studies are focused on identifying additional treatment options for patients who fail to exhibit a “good biochemical response” [25,28,29]. Several studies have validated the excessive deposition of BAs in the liver and considerable changes in the composition and diversity of the gut microbiota in patients with CLD [30,31,32,33,34]. Abnormal deposition of BAs has been observed to affect gut microbiota homeostasis through the enterohepatic circulation [35], and the gut microbiota, in turn, has been shown to affect the composition of the BA pool by metabolically breaking down BAs.

The past few years have witnessed a notable surge in studies investigating the role of BAs and gut microbiota in CLD [19,36,37,38]. Herein, we provide a summary of the existing research on the interactions between the gut microbiota and BAs in CLD. This review may contribute to our understanding of the novel mechanisms of BA signaling networks as potential therapeutic targets for CLD.

## 2. BA Homeostasis

### 2.1. Synthesis of BAs

**Figure 1:**
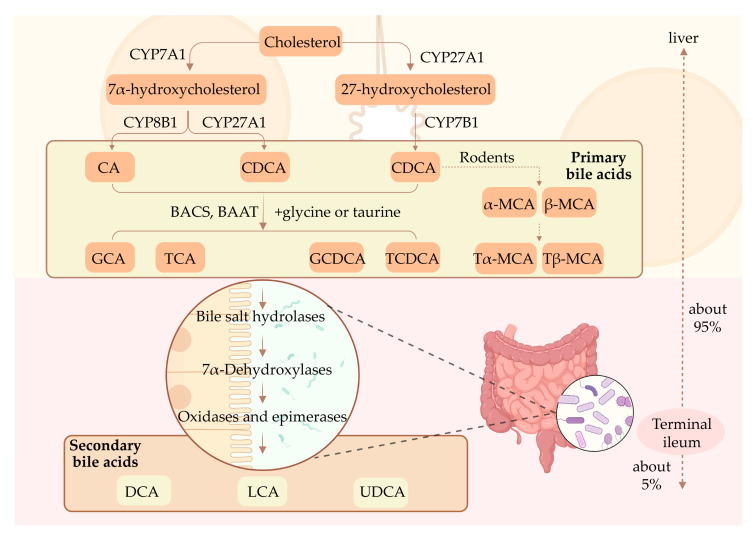
Bile acid synthesis and enterohepatic circulation. Schematic representation of the synthetic pathways of primary bile acids in hepatocytes and metabolism of secondary bile acids in the intestine. About 95% of the BAs are reabsorbed via the portal vein, whilst about 5% are reabsorbed through passive diffusion.

BAs are synthesized by cytochrome P450 (CYP)-mediated cholesterol catabolism in pericentral hepatocytes via two main biosynthetic pathways (Figure 1) [39]. The “classical” pathway begins with cholesterol 7α-hydroxylase (CYP7A1) acting on hepatic cholesterol, resulting in the generation of 7α-hydroxycholesterol, which is then subsequently catalyzed by CYP8B1 and CYP27A1, resulting in the formation of primary BAs, namely, cholic acid (CA) and chenodeoxycholic acid (CDCA), respectively [40]. In rodent livers, the majority of CDCA is transformed into alpha-muricholic acid and beta-muricholic acid [41]. The “alternative” pathway begins with sterol 27-hydrolase (CYP27A1), a mitochondrial CYP, hydroxylating cholesterol side chains, resulting in the generation of CDCA through 7α-hydroxylation of 27-hydroxycholesterol via CYP7B1 [42]. BA-coenzyme A (BA-CoA) synthetase and BA-CoA: amino acid *N*-acyltransferase then mediate the conversion of the primary BAs integrated with the amino acid taurine or glycine into taurocholic acid (TCA), taurochenodeoxycholic acid, glycine cholic acid (GCA), and glycochenodeoxycholic acid [16].

### 2.2. Enterohepatic Circulation of BAs

Primary BAs synthesized in the liver or absorbed into the enterohepatic circulation are actively transported to the bile canaliculus by the bile salt export pump (BSEP) or multidrug resistance-associated protein 2 (MRP2) [43,44,45]. Following a meal, the gallbladder constricts and releases BAs into the intestine, which then aid in the emulsification and absorption of lipids in the small intestine. Through the action of gut microbiota, primary BAs are converted into secondary BAs, such as deoxycholic acid (DCA), lithocholic acid (LCA), and UDCA. Subsequently, a small portion of unconjugated BAs is reabsorbed through passive diffusion in the anterior small intestine, whereas 95% of the BAs are efficiently reabsorbed in the terminal ileum via apical sodium-dependent BA transporter (ASBT) [46] and then transported to the basolateral membrane through ileal BA-binding protein. Thereafter, the organic solute transporter-α/β (OSTα/β) facilitates the entry of BAs into the portal vein blood circulation [47]. Ultimately, BAs are reabsorbed into hepatocytes through the sodium taurocholate co-transporting polypeptide (NTCP), organic anion-transport polypeptide 1 (OATP1), and OATP4 in the liver [48]. This entire process is called enterohepatic circulation, and plays a crucial role in BAs homeostasis.

### 2.3. BA Signaling Receptors

BAs achieve intercellular communication through various receptors. BAs can directly activate nuclear receptors, such as FXR, pregnane X receptor (PXR), constitutive androstane receptor (CAR), and vitamin D receptor (VDR), as well as a few G protein-coupled receptors (GPCRs), including TGR5 and sphingosine-1-phosphate receptor 2 (S1PR2) [49,50].

FXR is the major receptor for both free and conjugated BAs, and is highly expressed in hepatocytes and the intestine [51]. The activation of FXR by BAs can inhibit the expression of the rate-limiting enzyme CYP7A1 and mediate the negative feedback regulation of BA synthesis. Studies have shown that intestinal FXR signaling activates fibroblast growth factor (FGF) 15/19, which circulates through the portal vein to the liver, binds to FGF receptors on the surface of hepatocytes, and inhibits gene transcription of CYP7A1 via the Jun *N*-terminal kinas-dependent pathway, thereby inhibiting BA synthesis in the liver [52,53]. Conversely, the activation of FXR in the liver induces the transcription of small heterodimer chaperones and inhibits liver receptor homolog-1 and hepatocyte nuclear factor-4α, resulting in transcriptional repression of CYP7A1 [54]. Additionally, FXR activation upregulates the expression of BA efflux transporters BSEP, MRP2, and OSTα/β [55,56,57], and downregulates the expression of BA uptake transporters ASBT and NTCP [58], thereby preventing BA accumulation in hepatocytes.

TGR5 is a type of BA-specific GPCR and is expressed in the intestinal epithelium, liver sinusoid endothelium, liver macrophages, and Kupffer cells [59,60]. The ligands of TGR5 are primarily LCA, DCA, CDCA, and CA, which play vital roles in the regulation of energy homeostasis and improvement of insulin sensitivity [61,62]. PXR, CAR, and VDR are closely correlated with nuclear receptors with similar functions in BA detoxification and clearance [63]. S1PR2 is primarily expressed in hepatocytes and has been shown to activate the extracellular regulated protein kinases1/2 and protein kinase B signaling pathways by binding to its ligands, i.e., conjugated BAs, in rodent hepatocytes [64,65].

## 3. Effect of Gut Microbiota on BA Metabolism

### 3.1. Gut Microbiota Can Alter the Composition of the BA Pool

Gut microbiota is considered a crucial factor in BA homeostasis and substantially affects the chemical properties of BAs. In detail, gut microbiota has been shown to alter the composition of the BA pool through dehydrogenation, dihydroxylation, and desulfurization under the action of various enzymes.

#### 3.1.1. Bile Salt Hydrolase (BSH)

The first step in secondary BA metabolism is the hydrolysis of the amide bond by BSH. BSH, an intracellular enzyme encoded by the *BSH* gene [66], is a metabolic product that is synthesized by microorganisms during their growth and proliferation. BSH is insensitive to oxygen, and the optimal pH for its activity lies between 5 and 6 [67,68]. BSH cleaves the amide bond linking the glycine and taurine moieties of the steroid-binding nucleus of bile salts, resulting in the release of unbound BAs; the gut microbiota subsequently metabolizes these BAs by 7α-dehydroxylation, ultimately resulting in the production of secondary and tertiary BAs [69,70].

BSH has been identified in various microbial species across multiple phyla, including *Clostridium*, *Bifidobacterium*, *Lactobacillus*, *Bacteroidetes*, and *Enterococcus*. The presence of BSH has also been demonstrated in the archaea of the human intestine [71]. The amino acids released by BSH serve as an energy source for specific bacterial species, and its activity may contribute to microbial bile resistance and colonization of the gastrointestinal environment [72].

#### 3.1.2. Hydroxysteroid Dehydrogenase (HSDH)

Humans produce BAs that contain alpha-oriented hydroxyl groups, and gut microbiota can facilitate the biotransformation of these BAs into harmful hydrophobic BAs, such as DCA. Pyridine nucleotide-dependent HSDHs can reversibly oxidize the 3-, 7-, and 12-hydroxyl groups of CA by producing oxo-BA intermediates, thereby facilitating the generation of more hydrophilic and less harmful BAs. The BA-recognizing HSDH enzyme exhibits regional and stereo-specific properties and can modify the hydroxyl group of steroid nuclei [73]. To date, HSDH activity has been observed across a variety of bacteria, including *Bacteroidetes*, *Eubacillus*, *Clostridium*, *Bifidobacterium*, *Lactobacillus*, *Streptococcus peptidis*, and *Escherella* [74,75,76]. Genes associated with the BA pathway, such as that of *12ɑ-HSDH*, *7ɑ-HSDH*, *3ɑ-HSDH*, and *3β-HSDH*, have also been reported [77,78].

#### 3.1.3. 7α-Dehydroxylation Enzymes

Using 7α-dehydroxylase, bacteria in the colon convert primary BAs CA and CDCA to DCA and LCA, respectively [79], both of which are partially reabsorbed in the terminal ileum and transported back to the liver. 7α-dehydroxylation is a net reduction process; therefore, it is considered to be a key electron acceptance reaction in the energy metabolism of dehydroxylated bacteria [80,81]. 7α-dehydroxylase is exclusively present in low-abundance anaerobic bacteria, and multiple steps are catalyzed by BA-inducible enzymes encoded on the *bai* gene cluster [82]. Most of the bacteria that express *bai* belong to the *Ruminococcus* genus. In *Clostridium*, the operons *bai*A–J of the *bai* gene have been sequenced, and the enzyme encoded by the *bai* gene has been identified. In detail, BA 7α-HSDH is encoded by the *baiE* gene, and 7β-HSDH may be encoded by the *baiI* gene [9,79,83,84].

#### 3.1.4. Other Enzymes

Esterified BAs may account for more than 25% of the total BA content in feces. The esterification of BAs makes these molecules more hydrophobic and insoluble and results in the reduction of their concentration in feces [85]. The bacterial genera responsible for BA desulphurization include *Clostridium* and *Gastrococcus*. However, the role of gut microbiota in esterification and desulfurization remains unclear [82].

### 3.2. Gut Microbiota Affects BA Metabolism through FXR Signaling Molecules

The gut microbiota is involved in the transformation of BAs and the regulation of BA reabsorption. Intestinal microbiota not only participate in the decoupling, dehydrogenation, and dehydroxylation of BAs, but also negatively regulate BA synthesis through the FXR–FGF15/19 pathway [86]. BAs are synthesized from cholesterol in the liver and further metabolized into secondary BAs by the gut microbiota. The activation of the nuclear receptor FXR in the ileum and liver modulates the negative feedback regulation of BA production.

Sayin et al. analyzed the composition of the BA pool of the entire enterohepatic system of sterile and conventionally fed (CONV-R) mice and validated that CONV-R mice exhibited significantly decreased levels of muricholic acid but not CA [87]. Intestinal microbiota regulates the expression of FGF15 in the ileum and cholesterol 7α-hydroxylase (CYP7A1) in the liver through FXR-dependent mechanisms. Sun et al. conducted metagenomic and metabolomic analyses on stool samples from patients newly diagnosed with type 2 diabetes (T2D) [88]. The study revealed that following three days of metformin treatment, the abundance of *Bacteroides fragilis* in the intestine was decreased, and the levels of BA mandeoxycholic acid (GUDCA) were increased. These alterations, coupled with the inhibition of FXR signaling in the intestine, suggest that manipulation of the *B. fragilis*–GUDCA–intestinal FXR axis may ameliorate metabolic dysfunction. In addition, another study involving hamsters fed a 60% high-fat diet (HFD) and treated with antibiotics showed that increased levels of intestinal TMCA and decreased secondary BA levels, attributable to the loss of intestinal microbiota [89], result in inhibition of FXR signaling in the intestine and improvement in metabolic disorders. These studies indicate that gut microbiota can affect BA metabolism through FXR signaling molecules, suggesting that FXR can be an important target for regulating BA homeostasis.

## 4. Effect of BAs on Gut Microbiota

The size and diversity of the BA pool can affect the intestinal microbiota. Studies have shown that CA can alter the composition of gut microbiota in rats at the phylum level and lead to an increase in the number of Firmicutes and a decrease in the number of Bacteroidetes [67]. Compared to secondary bile salt supplementation, primary bile salt supplementation in the diet of *Ctenopharyngodon idellus* has been shown to cause more fluctuations in the composition of biliary BAs [90]. Primary bile salt increases intestinal microbiota diversity and induces microbiota succession, whereas secondary bile salt increases the ratio of Firmicutes to Bacteroidetes. In humans, BAs have been identified as host factors that affect the composition of intestinal microbiota after birth. UDCA, GCA, and TCA have been significantly correlated with alterations in gut microbiome composition with age. Consistent with these findings, oral administration of TCA has been shown to enhance postnatal microbiota maturation in neonatal mice [91].

BAs have toxic effects on the gut microbiome. In addition to harming bacterial cell membranes, BAs can also affect protein conformation, resulting in protein misfolding or denaturation; additionally, they can induce DNA damage and activate DNA repair-related enzymes, as well as generate oxygen free radicals, thereby causing oxidative stress [92]. Intestinal bacteria can resist BA damage through an adaptive response, which may require several proteins, including those responsible for the maintenance of cell envelope structure and intracellular dynamic balance [92]. Furthermore, BAs can affect mucosal immune responses and the integrity of intestinal epithelial cells, thereby indirectly regulating the composition and diversity of microbial communities.

The effect of BAs on the composition of intestinal microbiota can also be modulated by FXR. Through FXR, BAs can upregulate the expression of inducible nitric oxide synthase and the expression and secretion of interleukin 18, thereby preventing the proliferation of intestinal microorganisms [93]. Inagaki et al. revealed that FXR inhibits bacterial overgrowth and mucosal damage in the ileum caused by bile duct ligation, activates genes involved in intestinal defense, and plays a crucial role in the protection of the distal small intestine against bacterial invasion [94]. In addition, studies have shown that the composition of the BA pool and fecal microbiota is different between FXR^-/-^ and wild-type mice [95]. The intestinal microbiota of FXR-deficient mice is characterized by an increase in the abundance of Bacteroidetes and a decrease in the abundance of Firmicutes, indicating that BAs can affect the composition of the gut microbiota through the FXR signaling pathway. Furthermore, a clinical study showed that obeticholic acid (OCA) inhibits endogenous BA synthesis, causes reversible induction of Gram-positive bacteria, and improves the performance of microbial genomic pathways associated with DNA synthesis and amino acid metabolism [96]. Animal experiments have shown that OCA-fed mice exhibit decreased levels of endogenous BAs and increased abundance of Firmicutes in the small intestine [96].

## 5. Effect and Mechanism of BA–Intestinal Microbiota Interaction in CLD

**Figure 2:**
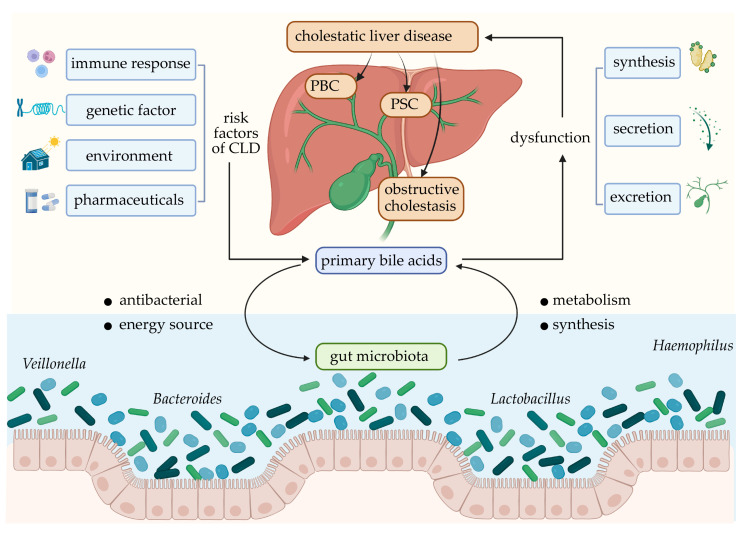
The interactions between bile acids and gut microbiota in cholestatic liver disease. Many risk factors, including immune response, genetic factor, environment, and pharmaceuticals, lead to dysfunction of synthesis, secretion, and excretion of primary bile acids, resulting in cholestatic liver diseases. Primary bile acids affect gut microbiota through antibacterial and energy source. The gut microbiota plays a role in the metabolism and synthesis of primary bile acids discharged into the intestine.

CLD is a type of hepatobiliary disease caused by immunological, genetic, and environmental factors, among others [25], wherein the generation, secretion, and excretion of bile inside and outside the liver are impaired, preventing the flow of bile into the duodenum and their entry into the blood. CLD can be classified as acute or chronic. Acute CLD may be caused by common bile duct stones and malignant tumors, whereas chronic CLD, which includes hereditary cholestatic disease, PBC, PSC, and secondary sclerosing cholangitis [35], involves extrahepatic and/or intrahepatic bile ducts [97]. Decreased microbial diversity was observed in both PBC and PSC patients [30,31]. In CLD, the main mechanisms by which abnormal BAs alter the gut microbiota include direct damage to the intestinal barrier, immune-mediated tissue assault, damage to bacterial cell membranes, promotion of antimicrobial peptide secretion through FXR binding, different resistance to BAs between species, etc. [32,35]. The interactions between BAs and gut microbiota play vital roles in CLD (Figure 2).

### 5.1. PBC

PBC is an autoimmune liver disease characterized by the presence of antimitochondrial antibodies and the progressive destruction of interlobular bile ducts [98]. There are significant differences in the composition and function of gut microbiota between patients with PBC and healthy individuals. Patients with PBC exhibit an imbalanced gut microbiota, with a decrease in the abundance of *Clostridium* and an increase in the abundance of *Lactobacillus* [99]. Patients newly diagnosed with PBC exhibit a significant increase in the abundance of multiple genera, such as *Haemophilus*, *Veillonella*, and *Clostridium*. UDCA treatment has been found to alter the composition of gut microbiota and partially improve the dysbacteriosis in patients with PBC [31].

The gut microbiota imbalance in PBC is associated with changes in the BA pool. The abundance of intestinal microbes in patients with PBC treated with UCDA varies according to bilirubin levels [100]. PBC is also associated with a decrease in the conversion rate of conjugated BAs to unconjugated BAs and primary BAs to secondary BAs. In patients with PBC, the concentration of secondary BAs, such as DCA and conjugated DCA, is negatively correlated with the increase in the abundance of intestinal microorganisms, such as *Veillonella* and *Klebsiella*, and positively correlated with microorganisms rich in healthy individuals, such as *Faecalibacterium* and *Oscillospira*. Following UDCA treatment, patients exhibit decreased levels of taurine-bound BAs, significantly increased levels of taurine-metabolizing bacteria *Bilophila* spp., and significantly decreased levels of FGF19 in the serum [101].

In PBC, BAs can promote liver regeneration by activating the FXR and TGR5 pathways [102]. The activation of the LXR pathway hinders the capillary formation by liver sinusoidal endothelial cells and reduces the generation of extracellular matrix to prevent fibrosis [103]. However, excessive elevation in the levels of BAs aggravates cellular necrosis and apoptosis and ultimately results in fibrosis [104]. The abnormalities in the composition of the BA pool and gut microbiota in PBC indicate the presence of a complex crosstalk between BA metabolism and BA pool composition and the composition of gut microbiota, which has crucial implications for the investigation of underlying mechanisms and therapy of PBC.

### 5.2. PSC

PSC is a chronic CLD and is characterized by biliary inflammation and periductal fibrosis [105]. PSC is typically progressive and may result in the development of complications such as cholestasis and liver failure. PSC is associated with ulcerative colitis and may be associated with cholangiocarcinoma. For patients who do not undergo liver transplantation, the median survival time since diagnosis is approximately 10 years.

Patients with PSC exhibit a decrease in the diversity of gut microbiota, with a significant increase in the abundance of *Enterococcus*, *Fusobacterium*, and *Lactobacillus*, independent of IBD or UDCA treatment. Furthermore, an operational classification unit of *Enterococcus* has been found to be positively correlated with elevated serum alkaline phosphatase (ALP) levels, indicating the severity of the disease [30]. The abundance of *Enterococcus* in the bile of patients with PSC is closely associated with the concentration of toxic secondary BA TCA [38]. The risk of colorectal cancer in patients with PSC associated with IBD (PSC–IBD) is very high [106]. The composition of the intestinal microbiota in patients with PSC–IBD is different from that of patients with IBD. In detail, the abundance of *Ruminococcus* and *Fusobacterium* bacteria is increased, and the abundance of *Dorea*, *Veillonella*, *Lachnospira*, *Blautia*, and *Roseburia* bacteria is reduced; additionally, a specific correlation between microbiota and BA content in feces has been elucidated [106].

In a PSC mouse model, the absence of intestinal microbiota has been shown to aggravate hepatobiliary diseases [107]. The imbalance in intestinal microbiota results in intestinal barrier dysfunction and increased bacterial translocation, which, in turn, promotes the progression of liver disease through NOD-like receptor protein 3 [34]. In mdr2^-/-^ mice and patients with PSC, *Lachnospiraceae* in feces are negatively correlated with the clinical severity of PSC, whereas *Enterococcus faecalis* and Enterobacteriaceae are positively correlated with the clinical severity of PSC. The inhibition of ileal BA transport alleviates antibiotic-induced progression of liver disease and reduces the total content of BA in the serum and liver [108]. In a genetic PSC mouse model, microbial depletion has been shown to impair FXR signal transduction, which then aggravates cholestatic liver injury. Subsequently, the lack of negative feedback control of BA synthesis results in an increase in BA concentration in the liver and impairment of the bile duct barrier function [109]. Intervention with *Prevotella* in PSC has been reported to enhance the FXR signaling pathway, leading to improvements in cholestasis and liver fibrosis [110].

### 5.3. Obstructive Cholestasis

Biliary obstruction typically refers to the obstruction of the biliary system [111], which results in the obstruction of bile flow from the liver to the intestine. The interruption of bile flow due to damage to the intrahepatic biliary system is commonly referred to as cholestasis. Cholestasis can manifest as abnormal liver function and can progress to jaundice and pruritus. The most common cause of biliary obstruction is common bile duct stones or gallstones.

A study involving 29 patients with gallstones and 38 normal individuals [112] revealed that, in patients with gallstones, the abundance of Proteobacteria is significantly increased, and the abundance of *Faecalibacterium*, *Lachnospira*, and *Roseburia* is decreased. Moreover, the diversity of biliary bacteria in patients with gallstones is significantly higher than that of intestinal bacteria, with most of the intestinal bacterial operational classification units being detected in the biliary tract. Similarly, another study revealed that in the feces of patients with gallstones, the total concentration of BAs is higher, microbial diversity is lower, the abundance of *Roseburia* is decreased, and the abundance of *Oscillospira* is increased [113]. Furthermore, cholecystectomy has been shown to lead to an increase in the abundance of Bacteroidetes in the feces.

Wu et al. simulated the processes of biliary obstruction, drainage, and bile reinfusion in a mouse model and found that, despite the occurrence of biliary obstruction, mice continued to produce secondary BAs, and their levels were increased in both the liver and serum. Following 12 days of biliary obstruction, the abundance of *Ruminococcus_1* was observed to be increased [114]. In another study, the fecal microbiota, which is rich in Desulfovibrionales, of patients with gallstones was transplanted into gallstone-resistant strains of mice to induce gallstone formation [115]. Desulfovibrionales affected the BA pool, increased the production of secondary BAs, promoted intestinal cholesterol absorption, and its metabolite H2S promoted FXR expression and inhibited CYP7A1 expression. In addition, mice harboring Desulfovibrionales showed the expression of cholesterol transporter Abcg5/g8, which promotes cholesterol bile secretion. A novel FXR agonist, namely, TC-100, has been shown to activate FXR in the intestinal–hepatic axis, reduce the size of BA pools of the serum and bile, and convert them into more hydrophilic BAs, thereby preventing intestinal mucosal damage. Furthermore, the ratio of Firmicutes to Bacteroidetes has been shown to gradually increase in TC-100-treated mice [116].

In general, in CLD, gut microbiota affects disease progression by interacting with BAs. Drugs that affect BA metabolism or alter intestinal microbiota may be used for the treatment of these diseases. A better understanding of the relationship between diseases and intestinal microorganisms is crucial for developing treatment strategies.

## 6. Potential Therapeutic Targets

Traditional interventions aimed at gut microbiota include prebiotics, probiotics, antibiotics, and fecal microbiome transplantation (FMT) [35]. Probiotics are widely involved in BA anabolism pathways, including that of CDCA, DCA, and LCA, and can regulate FXR signaling and the composition of the intestinal microbiota [117]; therefore, they may be used as potential adjuvant treatment for cholestasis. In a mouse model-based experiment, the probiotic *Lactobacillus rhamnosus* GG (LGG) was shown to inhibit liver BA synthesis and enhance BA excretion by activating the intestinal FXR–FGF15 signaling pathway, thereby preventing liver injury and fibrosis in mice [118]. Additionally, a series of animal experiments have elucidated the great potential of LGG in the treatment of intrahepatic cholestasis of pregnancy [119] and cholestatic drug-induced liver injury [120]. In an animal experiment, antibiotic treatment partially eliminated increased cholestasis observed in triggering receptor expressed on myeloid cells-2 (TREM-2)-deficient mice after bile duct ligation (BDL) [121]. The experiment also demonstrated that TREM-2, as a negative regulator of inflammation, may be a potential therapeutic target for CLD. In another research based on a murine model of PSC, early FMT reduced mortality in multidrug-resistant 2-deficient (*mdr2-/-*) mice [108]. Other potential therapeutic targets based on BA signaling for CLD are described below in detail.

### 6.1. FXR Agonist

The activation of FXR inhibits the synthesis of BAs in hepatocytes, increases the efflux of BAs from hepatocytes, thereby reducing the exposure of hepatocytes to BAs, and has become a key therapeutic target for cholestasis [122]. At present, drugs targeting FXR activation include steroid FXR agonists, such as OCA, and non-steroidal FXR agonists, such as cilofexor, tropifexor, MET409, EDP-305, and EYP001. OCA has been approved by the US Food and Drug Administration [123] and China Food and Drug Administration as the first steroid FXR agonist for treating patients with CLD who are not fully responsive to or intolerant of UDCA [124]. In March 2023, the preliminary results of a phase IV trial (NCT02308111) were released, which was terminated early on the recommendation of the Data Monitoring Committee, as the design of the post-marketing study was not feasible. However, adverse events (AEs), such as pruritus, and concerns about long-term cardiovascular safety, with elevated levels of low-density lipoprotein cholesterol, may affect the promotion of OCA as a clinical drug [50].

Non-steroidal FXR agonists are currently topics of intense research, with many related drugs having been developed. Cilofexor is an effective non-steroidal FXR agonist. A new phase II trial (NCT02943460) involving 52 patients with PSC who were treated with cilofexor for 96 weeks showed that cilofexor improved liver biochemistry and biomarkers of cholestasis; specifically, the treatment led to a reduction in the levels of γ-glutamyl transpeptidase (GGT), alanine aminotransaminase (ALT), aspartate aminotransferase (AST), and other indicators [125,126]. Besides that, a phase III, randomized, and double-blind clinical trial (NCT03890120) aimed at assessing the potential of cilofexor in reducing the risk of fibrosis progression among non-cirrhotic adults diagnosed with PSC is currently underway, with results yet to be reported. Tropifexor is another non-steroidal FXR agonist. Its efficacy was investigated in a phase II clinical trial (NCT02516605) involving 61 patients with PBC who showed an inadequate response to UDCA. The results showed that the cholestatic markers in the tropifexor group were improved compared with the placebo group [127], with pruritus as the most common AE. To date, studies on MET409, EDP-305, and EYP001 have primarily focused on nonalcoholic steatohepatitis (NASH) and hepatitis B, and only a few studies have focused on CLD [128].

### 6.2. Peroxisome Proliferator-Activated Receptor (PPAR) Agonists

In recent years, bezafibrate, a PPARα agonist, elafibranor, a PPARα/δ agonist, seladelpar, a PPARδ agonist, and saroglitazar, a PPARα/γ agonist, have been investigated for the treatment of CLD. Several studies have demonstrated that the combination of UDCA and bezafibrate, a type of fibrate, can improve biochemical measures and long-term outcomes in patients with PBC [129,130]. Importantly, a study conducted in the Netherlands showed that bezafibrate reduces the intensity of pruritus, which was associated with a decrease in serum ALP levels, in patients with PSC and PBC [131]. The specific mechanism underlying the alleviation of pruritus may involve the mitigation of liver cholestasis, cytokine-mediated biliary inflammation, and fibrosis [132]. A clinical trial on seladelpar for the treatment of PSC was once withdrawn because of suspected liver damage in a few patients who had received the drug. Nevertheless, in 2022, a 52-week phase II clinical trial (NCT02955602) demonstrated that seladelpar treatment improves ALP in patients with PBC who are unresponsive or intolerant to UCDA [133]. In a 12-week phase II clinical trial [134], elafibranor was observed to reduce ALP, bilirubin, and GGT levels in patients with PBC who were unresponsive to UDCA. In addition, a phase II clinical trial on lanifibranor, a pan-PPAR agonist, for the treatment of experimental advanced chronic liver disease [135], showed that lanifibranor improved portal hypertension and hepatic fibrosis, indicating that it may have the potential to be used for CLD treatment.

### 6.3. FGF19 Analogs

Analogs of FGF19, such as NGM282 (aldafermin), have been developed; FGF19 is expressed in the terminal ileum, reaches the liver through the intestinal-hepatic circulation, and binds to the FGFR4/βKlotho receptor complex [128]. Aldafermin is a non-carcinogenic engineered variant of the human hormone FGF19. Although, the initial purpose of developing FGF19 analogs was primarily to improve hyperglycemia in patients with T2D [136], NGM (NGM Biopharmaceuticals, Inc.) In recent years, NGM completed phase II clinical studies of NASH and PSC. In a phase II clinical trial involving 62 patients with PSC, liver fibrosis biomarkers were significantly reduced in the NGM282 group, with no differences in the ALP levels following 12 weeks of treatment with NGM282. Aldafermin inhibits the synthesis of BAs and protects the liver from cholestasis by inhibiting CYP7A1 in the liver [137]. However, the primary concerns in the development and application of these drugs are gastrointestinal symptoms and the potential risk of liver cancer.

### 6.4. Others

In addition to the popular drugs described in the previous sections, many other types of drugs exist. A variety of PXR agonists exists, such as rifampicin, which is an effective human PXR agonist. Currently, the use of rifampicin and the PPAR agonist bezafibrate for the treatment of pruritus in patients with PBC and PSC has been supported by empirical evidence [132]. FXR/TGR5 dual agonists, such as INT-767, have been reported to have therapeutic effects on metabolic diseases, such as NASH and diabetes [138], and to inhibit hepatitis B virus infection [139]. Phase II clinical trials were conducted to explore the therapeutic effect of simtuzumab, a monoclonal antibody targeting lysyl oxidase-like 2 [140], and cenicriviroc, a dual chemokine receptor-2 (CCR2)/CCR5 chemokine receptor antagonist [128], on PSC; however, the results were not significant.

In recent years, the pharmacological properties of traditional Chinese medicine have been extensively studied. Traditional Chinese medicines targeting BA metabolic pathways have gradually emerged. For example, lignans, which are bioactive components isolated from Schisandrae chinensis Fructus, have been elucidated to play protective roles by activating the PXR signaling pathway in cholestatic liver injury [120].

## 7. Conclusions and Perspectives

The interaction between BAs and gut microbiota is associated with the progression and prognosis of CLD. The relationship among them is not one-way, but highly intertwined in a network. Much remains to be discovered about the specific mechanisms by which the gut microbiota and BAs are involved in pathophysiological processes. Treatments targeting BA signaling pathways and gut microbiota have proved successful, but more research is needed to further explore new therapeutic targets and the molecular mechanisms of symptoms remission in CLD, and more clinical trial evidence is needed.

## Data Availability

Not applicable.
